# Functional Characterization of the SCN5A p.D372H Variant Associated with Brugada Syndrome

**DOI:** 10.3390/biomedicines14030582

**Published:** 2026-03-05

**Authors:** Xianghuan Xie, Yunqi He, Yanghui Chen, Zhiqiang Li, Yang Sun, Guangzhi Chen

**Affiliations:** 1 Division of Cardiology, Department of Internal Medicine, Tongji Hospital, Tongji Medical College, Huazhong University of Science and Technology, 1095# Jiefang Ave., Wuhan 430030, China; 2 Hubei Key Laboratory of Genetics and Molecular Mechanisms of Cardiological Disorders, Wuhan 430030, China; 3 Department of Medical Ultrasound, Tongji Hospital, Tongji Medical College, Huazhong University of Science and Technology, 1095# Jiefang Ave., Wuhan 430030, China; 4 Department of Cell Biology, SUNY Downstate Health Sciences University, Brooklyn, NY 11203, USA

**Keywords:** Brugada syndrome, Brugada, genetics, genes, SCN5A, variant

## Abstract

**Background:** Brugada syndrome (BrS) is a genetic cardiac arrhythmia disorder inherited in an autosomal dominant manner, characterized by ST-segment elevation in the right precordial leads (V1–V3) on electrocardiograms (ECGs). This syndrome predominantly affects young individuals with structurally normal hearts and significantly increases the risk of ventricular arrhythmias and sudden cardiac death (SCD). The most common genotype found among BrS patients is caused by variants in the SCN5A gene, which lead to a loss of function of the cardiac sodium channel Nav1.5 by different mechanisms. **Methods:** Plasmids containing SCN5A were constructed using PCR and site-directed mutagenesis to create the D372H variant. HEK293 cells were cultured and transfected with the WT, D372H, or a combination of both plasmids. Patch-clamp recordings assessed sodium current characteristics. Confocal microscopy visualized channel localization. Quantitative RT-PCR was used to analyze mRNA expression levels, while Western blot evaluated protein expression using specific antibodies. **Results:** In HEK293 cells expressing the D372H mutant, functional assays revealed a near-complete loss of sodium currents. Co-transfection of WT and D372H plasmids resulted in a significant reduction in current density compared with WT alone, while activation, inactivation, and recovery kinetics were unaffected. In addition, both the mutant protein and protein expressed in co-transfected cells exhibited reduced fluorescence intensity, indicating decreased expression levels. These findings were further supported by Western blot and RT-qPCR analyses. **Conclusions:** In summary, our findings indicate that the D372H variant produces a marked reduction in Nav1.5 function through reduced sodium current density and decreased channel expression. Given its critical position within the DI-pore loop, this defect is expected to markedly diminish the inward sodium current necessary for normal depolarization. Such impaired excitability—particularly relevant in the right ventricular outflow tract—may accentuate regional differences in repolarization and create conditions that favor reentrant activity. These findings provide mechanistic insights into how the p.D372H variant alters Nav1.5 channel function in vitro and offer functional evidence that may assist in interpreting its potential relevance to Brugada syndrome.

## 1. Introduction

Brugada syndrome (BrS) is a genetic cardiac arrhythmia syndrome inherited in an autosomal dominant manner, characterized by distinctive electrocardiogram (ECG) changes, specifically ST-segment elevation in the right precordial leads (V1–V3) [1]. This condition primarily affects young individuals with structurally normal hearts and is associated with an increased risk of ventricular arrhythmias and sudden cardiac death (SCD) [2,3]. BrS is estimated to account for 4–12% of all sudden death cases and approximately 20% of sudden cardiac deaths in individuals under 50 years of age who have no identifiable structural heart disease [4,5]. The prevalence of BrS ranges from 1 in 2000 to 1 in 5000, with symptoms typically emerging in adulthood; the average age of SCD is reported to be 41 ± 15 years [6].

To date, more than 500 variants have been discovered in over 40 genes that may be associated with BrS [7]. These variants primarily involve genes that encode sodium [8], potassium [9], and calcium [10] channels, as well as related regulatory proteins. Nonetheless, most of these genetic variants remain functionally uncharacterized. At present, the *SCN5A* gene, encoding the cardiac sodium channel Nav1.5, is the only gene conclusively associated with BrS. Variants in *SCN5A* account for fewer than 30% of cases, leaving more than 60% of patients without an identified genetic cause [11,12].

The cardiac sodium channel α-subunit Nav1.5, encoded by the *SCN5A* gene, plays a critical role in generating action potentials in cardiac muscle. Nav1.5 is composed of 2016 amino acids arranged into four homologous domains (DI–DIV) [13,14]. Each of these domains contains six transmembrane α-helices (S1–S6), which are interconnected by intracellular loops. The voltage-sensing apparatus is primarily formed by the S1–S4 segments, which incorporate positively charged amino acids essential for channel activation. In contrast, the S5 and S6 segments, along with the S5–S6 loops, collectively create the channel’s pore and the selectivity filter for sodium ions (Na^+^). While *SCN5A* variants linked to Brugada syndrome (BrS) are dispersed throughout the entire channel structure, a combination of structural and functional analyses has identified that certain variants in specific regions are associated with distinct biophysical defects.

**Figure 1:**
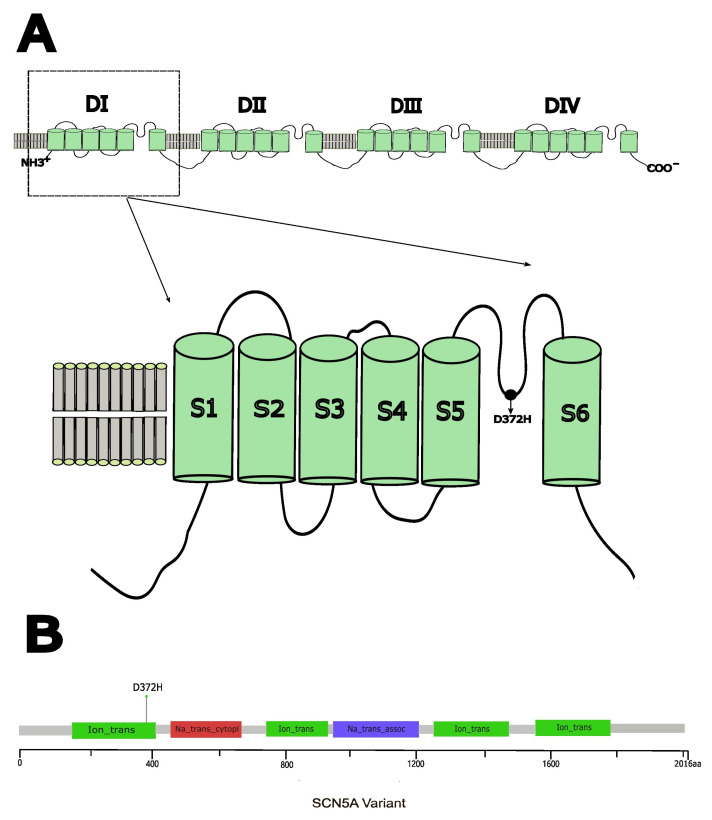
(**A**) Schematic representation of the cardiac voltage-gated Na^+^ channel α-subunit (Nav1.5) showing the position of the D372H variant in the linker between segments 5 and 6 in domain I (DIS5–S6). (**B**) Needle plot of the D372H variant in the *SCN5A* gene at the protein level across BrS patients.

In our previous research, we discovered a novel variant, NM_198056.2: c.1114G > C (p.D372H) [15], situated in domain I of the Nav1.5 protein, specifically within the pore-forming intramembrane region between the S5 and S6 transmembrane segments (Figure 1) [16,17]. This variant was found in a 48-year-old male patient admitted with syncope, exhibiting a spontaneous type 1 Brugada syndrome ECG pattern characterized by concealed ST-segment elevation. The patient also suffered from sleep apnea syndrome accompanied by paroxysmal second-degree type I atrioventricular block, indicating arrhythmic events. Family history revealed the patient’s mother had a history of syncope and sudden cardiac death at age 50. Genetic testing of the patient’s siblings and daughter showed no variant and no symptoms, whereas his son carried the same variant, presenting with first-degree atrioventricular block and mild ST-segment elevation. According to the American College of Medical Genetics and Genomics (ACMG) guidelines, the classification of a variant as pathogenic can be supported by well-established functional studies showing a deleterious effect (PS3 criterion). The p.D372H variant has been previously described in case reports as a potentially disease-associated variant; however, functional studies have not been performed to substantiate its pathogenicity and clinical significance. In 2020, Tadashi Nakajima and colleagues identified the *SCN5A* W374G variant, which is located in the pore loop of Domain I, in cases of severe Brugada syndrome (BrS) and is in close proximity to the D372H variant. Their functional studies indicated that the W374G variant led to a reduced current density, likely due to trafficking defects, and a depolarizing shift in steady-state activation (SSA), highlighting that variants in this region can be associated with altered channel function [18]. These findings suggest that variants within this domain significantly disrupt Na^+^ channel function; however, the impact of the D372H variant on channel activity has not been thoroughly investigated. The aim of this study was to investigate the functional consequences of the *SCN5A* p.D372H variant identified in a patient with Brugada syndrome. Using heterologous expression in HEK293 cells combined with electrophysiological recordings, confocal imaging, and expression analyses, we evaluated the effects of this variant on Nav1.5 channel function and protein expression. These experiments were designed to determine whether the p.D372H variant alters sodium channel properties in a manner consistent with previously reported Brugada syndrome-associated *SCN5A* variants, thereby providing functional evidence to aid interpretation of its potential clinical relevance.

## 2. Materials and Methods

This study employed an in vitro expression system to examine the functional effects of the *SCN5A* p.D372H variant on Nav1.5 channel properties. Electrophysiological, imaging, and molecular approaches were used to assess alterations in sodium current characteristics and channel expression.

### 2.1. Plasmid Construction

According to the gene sequence of *SCN5A* provided by PubMed (reference sequence NM_198056), we designed primers for the flanks of introns to amplify the entire *SCN5A* gene by polymerase chain reaction (PCR) and incorporated GFP to create a fusion gene. Plasmid encoding *SCN5A* (Nav1.5) was generated using a Vazyme site-directed mutagenesis kit (Vazyme Biotech Co., Ltd., Nanjing, China). according to the manufacturer’s instructions. All plasmid constructs were verified by Sanger sequencing across the entire coding region to confirm the presence of the intended variant and absence of unwanted sequence changes. The cloning of the relevant fragment of *SCN5A* variant (D372H) was generated using overlap extension PCR and inserted as an EcoR1/HindIII fragment into the EcoR1/HindIII site of *SCN5A* cDNA of pcDNA3.1-EGFP-3×FLAG, which AuGCT Biotechnology provided. The head and tail primers used are as follows:

5′-CCAAGCTGGCTAGCGTTTAAACTTAAGCTTGCCACCATGATTCCTGGTAACCGAA-3′

5′-GATGGTAGTAGAGGGATGTGGGTGCCGCTGAGAATTCTGCAGATATCCAGCACAGTGGTGCG-3′

All constructs were purified using Qiagen columns (QIAGEN Inc., Hilden, Germany). The cDNA was sequenced to confirm the presence of the correct fragment, containing the variant.

### 2.2. Cell Culture and Transient Expression in HEK293 Cells

HEK293 cells were cultured in Dulbecco’s modified Eagle’s medium (DMEM; BOSTER, Pasching, Wuhan, China) supplemented with 10% fetal bovine serum (FBS; BOSTER, Pasching, Wuhan, China) and 1% penicillin–streptomycin, and maintained at 37 °C in a humidified atmosphere containing 95% air and 5% CO_2_.

For transfection, cells were seeded in 6-well plates at approximately 60–70% confluence and transfected using HighGene transfection reagent (5 μL per well) with plasmids encoding pcDNA3.1-EGFP-3×FLAG wild-type *SCN5A* (WT, 2 μg), pcDNA3.1-EGFP-3×FLAG-D372H mutant *SCN5A* (MUT, 2 μg), or a 1:1 combination of WT and MUT plasmids (WT+MUT, 1 μg each; total DNA 2 μg).

Following transfection, cells were incubated for 48 h at 37 °C before being subjected to patch-clamp electrophysiological recordings, confocal imaging, and biochemical analyses.

### 2.3. Electrophysiology

Whole-cell patch-clamp recordings were performed on HEK293 cells transiently transfected with *SCN5A*-WT or *SCN5A*-D372H. Sodium currents were recorded using a patch-clamp amplifier (HEKA Elektronik, Lambrecht/Pfalz, Germany) and analyzed with PatchMaster software, version 2x73 (HEKA Elektronik, Lambrecht/Pfalz, Germany). Patch pipettes were pulled from borosilicate glass capillaries with a resistance of 2–5 MΩ when filled with internal solution. Series resistance was compensated by 70–80%, and cells with leak currents > 10% of peak current were excluded from analysis. The extracellular solution contained the following components (in mM): 140 NaCl, 3.5 KCl, 1 MgCl_2_, 2 CaCl_2_, 10 glucose, 10 HEPES, and 1.25 NaH_2_PO_4_ (pH 7.4). The pipette (intracellular) solution contained the following components (in mM): 50 CsCl, 10 NaCl, 10 HEPES, 60 CsF, and 20 EGTA (pH 7.2).

### 2.4. Sodium Current Characteristics

The membrane was held at −120 mV, and test voltages ranging from −80 mV to +60 mV were applied for 100 ms. Representative schematic traces and voltage-clamp protocols illustrating sodium channel activation, steady-state inactivation, and recovery from inactivation under normal conditions are shown in Supplementary Figure S1. Sodium current amplitudes were converted to conductance and normalized to the maximal conductance to generate the steady-state activation curve. The data were fitted with the Boltzmann equation *y* = 1/[1 + exp (−(*x* − *V*_1/2_)/*κ*)] to determine the half-activation voltage (*V_1/2_*) and the slope factor (*κ*).

A double-pulse protocol was employed, with the membrane held at −120 mV and conditioning voltages ranging from −140 mV to +20 mV applied for 100 ms, followed by a 50 ms test pulse at −20 mV. Steady-state inactivation curves were obtained by normalizing the peak current during the test pulse to the maximal current and fitting the data with the Boltzmann equation to derive *V*_1/2_ and *κ*.

Recovery from inactivation was assessed using a triple-pulse protocol. Cells were held at −120 mV, depolarized to −20 mV for 100 ms to induce channel inactivation, and then allowed to recover at −120 mV for intervals ranging from 1 to 1000 ms before a second 50 ms pulse at −20 mV. Recovery curves were generated by normalizing the peak currents of the second pulse to the maximal current. Data were fitted using either a double-exponential function *y* = 1 − *A*_1_ exp(−*x*/*τ*_1_) − *A_2_* exp(−*x*/*τ*_2_) or a single-exponential function *y* = *A*_1_ exp(−*x*/*τ*_1_) + *B*, where *A*_1_ and *A*_2_ represent the relative contributions of the fast and slow recovery components, and *τ*_1_ and *τ*_2_ denote their respective time constants. All voltage-clamp protocols and analyses were performed using the same acquisition settings across experimental groups to ensure comparability.

### 2.5. Confocal Microscopy

To determine the subcellular localization of NaV1.5 channels, including the wild-type (WT) and the D372H mutant, in HEK293 cells, confocal microscopy was performed on cells transfected with GFP-tagged NaV1.5 constructs. HEK293 cells were transfected with plasmids encoding GFP-tagged NaV1.5 and incubated for 48 h. After incubation, cells were washed three times with phosphate-buffered saline (PBS) to remove residual medium and unbound plasmid. The cells were then fixed with 4% formaldehyde for 30 min at room temperature, followed by three additional washes with PBS. To visualize the plasma membrane and assess membrane localization of NaV1.5, cells were incubated with the lipophilic membrane dye DiI (BOSTER, Pasching, Wuhan, China) according to the manufacturer’s instructions. Subsequently, nuclear staining was performed using DAPI (BOSTER, Pasching, Wuhan, China) for 15 min. After staining, cells were washed with PBS and mounted on glass coverslips. A microscope (Leica, Microsystems, Wetzlar, Germany) was used to visualize the localization of the fluorescent channels. Images were acquired using identical laser power, detector gain, and exposure settings across groups. Fluorescence intensity was quantified using ImageJ software, version 1.54g (National Institutes of Health, Bethesda, MD, USA). with background subtraction performed before analysis. At least three independent transfections were analyzed.

### 2.6. Quantitative Real-Time RT-PCR Analysis

To evaluate the effect of the *SCN5A* p.D372H variant on *SCN5A* mRNA expression, total RNA was extracted from transfected cells using TRIzol reagent (TaKaRa, Dalian, China) according to the manufacturer’s instructions. RNA concentration and purity were assessed spectrophotometrically. Complementary DNA (cDNA) was synthesized using a reverse transcription kit (Vazyme Biotech Co., Ltd., Nanjing, China). Quantitative real-time PCR (qPCR) was performed using a qPCR 900 system and ChamQ Universal SYBR qPCR Master Mix (Vazyme Biotech Co., Ltd., Nanjing, China). Each reaction contained 2 μL cDNA template, 10 μL SYBR Green master mix (including ROX), and 200 nM of each forward and reverse primer in a final volume of 20 μL. The thermal cycling conditions were as follows: initial denaturation at 95 °C for 30 s, followed by 40 cycles of denaturation at 95 °C for 10 s and annealing/extension at 60 °C for 30 s. Relative mRNA expression levels were calculated using the 2^−ΔΔCt^ method, with tubulin serving as the internal reference gene. All samples were analyzed in technical triplicate, and at least three independent biological replicates were performed. Each sample was analyzed in technical triplicate, and at least three independent biological replicates were performed. The primer sequences used are listed below:

*SCN5A*, 5′-GTCTCAGCCTTACGCACCTT-3′ (forward);

5′-GGCAGAAGACTGTGAGGACC-3′ (reverse);

TUBULIN, 5′-GACAAGACCATTGGGGGAGG-3′ (forward);

5′-ACAGGCAGCAAGCCATGTAT-3′ (reverse).

To evaluate whether the D372H variant affects *SCN5A* transcript stability, mRNA decay was assessed following transcriptional inhibition. Forty-eight hours after transfection (WT or D372H), cells were treated with actinomycin D (5 μg/mL) to block new RNA synthesis. Total RNA was collected at 0, 1, 2, 4, and 6 h after treatment. *SCN5A* mRNA levels were quantified by RT-qPCR and normalized to tubulin. Relative mRNA remaining was calculated by setting the 0-h time point as 1.0. Decay curves were fitted using nonlinear regression (one-phase exponential decay model) in GraphPad Prism, version 9.1.0 (GraphPad Software, San Diego, CA, USA).

### 2.7. Western Blot

To assess the expression levels of Nav1.5 channels, Western blot analysis was performed on transfected HEK293 cells. The cells were seeded in six-well plates and incubated for 48 h. Following this incubation period, IP lysate buffer was added to each well, and the plates were placed on a shaker and mixed at 4 °C for 15 min to extract total proteins. Protein concentrations were determined using the BCA quantification method. Subsequently, the proteins were separated using SDS-PAGE with a gradient of 4% to 12% (Boster, Wuhan, China). Equal amounts of total protein (20–30 µg) were loaded per lane. After electrophoresis, the proteins were transferred to a nitrocellulose membrane. The membrane was then incubated with primary antibodies against Nav1.5 (1:1000, Cat# 23016-1-AP, Proteintech, Wuhan, China) and Tubulin (1:000, Cat# A03989-1, Boster, Wuhan, China) to detect the target protein and serve as a loading control, respectively. Following incubation with the primary antibodies, the membrane was washed three times with TBST (Tris-buffered saline with Tween 20) to remove unbound antibodies. The membrane was then incubated with a goat anti-rabbit secondary antibody ((1:20,000, Cat# 926-32111, LI-COR Biosciences, Lincoln, NE, USA) to visualize the protein bands. Band intensities were quantified using ImageJ and normalized to tubulin. All experiments were repeated independently at least three times.

### 2.8. Statistical Analysis

All data are presented as mean ± SD (standard deviation). Statistical analyses were performed using SPSS version 17.0 (SPSS, Chicago, IL, USA) or GraphPad Prism (version 9). Data distribution was assessed for normality before applying parametric tests. Comparisons between two groups were performed using unpaired two-tailed Student’s *t*-tests when data were normally distributed. For comparisons involving more than two groups, one-way analysis of variance (ANOVA) followed by appropriate post hoc tests was used. A *p* value < 0.05 was considered statistically significant. At least three independent biological replicates were performed for each experiment.

This study was conducted in a heterologous HEK293 expression system, which allows controlled assessment of channel properties but does not fully replicate the cellular environment of human cardiomyocytes. Therefore, the observed functional effects reflect channel behavior under in vitro conditions and may not capture the full complexity of cardiac electrophysiology in vivo.

## 3. Results

### 3.1. D372H Variant Significantly Impairs Nav1.5 Sodium Current Expression

**Figure 2:**
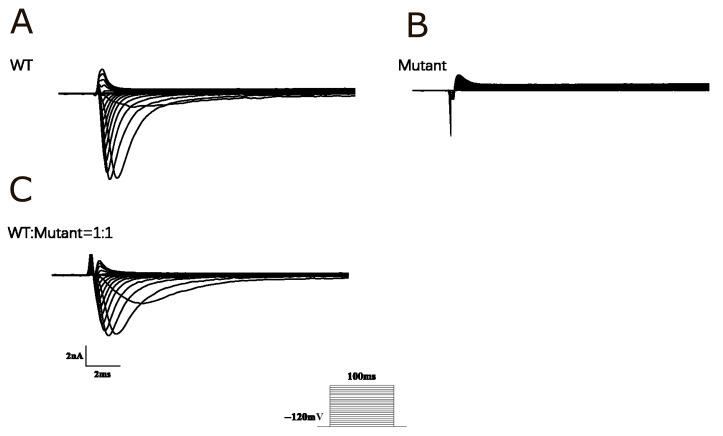
Functional analysis of Nav1.5 channels. (**A**) Current traces from HEK293 cells expressing wild-type (WT) Nav1.5 channels show robust sodium currents, indicating normal channel function with rapid activation and inactivation. (**B**) In contrast, traces from cells expressing the D372H mutant reveal nearly absent sodium currents, indicating substantially impaired channel function. (**C**) Current traces from cells co-expressing WT and D372H mutant Nav1.5 channels at a 1:1 ratio demonstrate a reduction in overall current compared to cells transfected with 2 μg WT plasmid alone, consistent with the near absence of sodium current produced by the D372H mutant channels. The I-V curve analysis shows a decrease in current density in the co-expression condition, reflecting the dilution effect of the nonfunctional D372H channels.

To investigate the pathophysiological mechanisms underlying the phenotype of Brugada syndrome, Nav1.5 channels were transiently expressed in HEK 293 cells, and the functional impact of the D372H variant was assessed using whole-cell patch-clamp recordings. In cells transfected with wild-type (WT) Nav1.5 channels, robust sodium currents were observed. In contrast, sodium currents were almost completely abolished in cells transfected with the D372H mutant. To further explore the effects of this variant, we designed a subsequent patch-clamp experiment where equal amounts of wild-type (WT) and D372H plasmids were co-transfected into HEK293 cells at a 1:1 ratio. Specifically, 1 μg of WT plasmid (approximately 751.7 ng/μL) and 1 μg of D372H plasmid (approximately 791 ng/μL) were used per well, keeping the total DNA amount consistent with single-transfection experiments. Whole-cell patch-clamp recordings were conducted after transfection to assess the resulting sodium currents. Analysis of the currents recorded from WT-expressing cells revealed a significant reduction in current density after co-expression with the D372H variant, as demonstrated by the I-V curve (Figure 2). This finding indicates that *SCN5A*-WT/p.D372H (1:1) co-expression leads to the down-regulation of channel activity, with statistical significance observed between the groups (*p* < 0.05).

**Figure 3:**
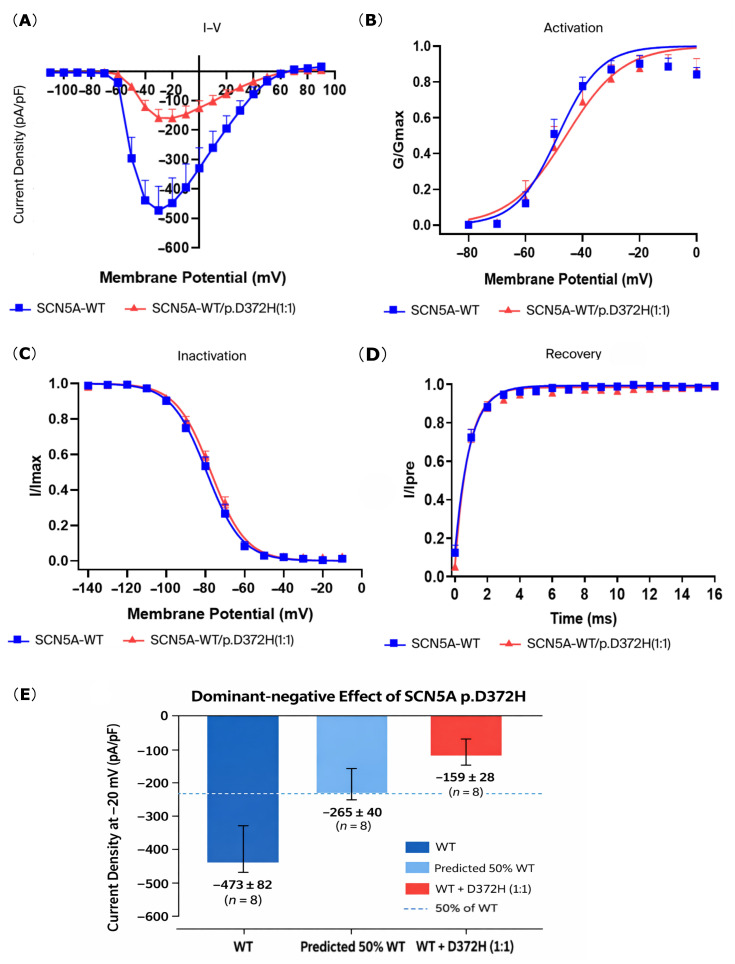
Co-expression of *SCN5A*-WT and *SCN5A*-D372H significantly reduced sodium current density but did not significantly alter activation, inactivation, or recovery kinetics. (**A**) The I–V relationship shows sodium current density in HEK293 cells expressing either *SCN5A*-WT (blue squares) or *SCN5A*-WT/p.D372H (red triangles). Peak current density was significantly reduced in the *SCN5A*-WT/p.D372H group compared with *SCN5A*-WT. (**B**) Steady-state activation curves of sodium channels. Although the D372H mutant showed a slight rightward shift compared to WT, the differences in half-activation voltage (*V_1/2_*) and slope factor (*κ*) were not statistically significant. (**C**) Steady-state inactivation curves demonstrate that the inactivation kinetics of *SCN5A*-WT and *SCN5A*-WT/p.D372H were comparable, indicating no significant effect of the variant on channel inactivation. (**D**) Recovery from inactivation curves. Both groups exhibited similar recovery kinetics, showing that the D372H variant did not significantly impact recovery dynamics. (**E**) Dominant-negative effect of the *SCN5A* p.D372H variant. Bar graph showing peak sodium current density at −20 mV in HEK293 cells expressing WT Nav1.5, the predicted 50% haploinsufficiency level, and WT+D372H (1:1). The observed current density in WT+D372H cells was significantly lower than the predicted 50% level, indicating a dominant-negative effect of the D372H variant on WT channel function.

As summarized in Table 1, co-expression of *SCN5A*-WT and p.D372H (1:1) resulted in a marked reduction in peak sodium current density at −20 mV compared with WT alone. The I–V relationship (Figure 3A) further demonstrated attenuated inward sodium currents across the tested voltage range in the WT+D372H group. In contrast, the voltage dependence of activation and inactivation was largely preserved, with no significant differences in *V*_1/2_, *κ*, steady-state inactivation, or recovery from inactivation kinetics between WT and WT+D372H channels (Table 1 and Figure 3B–D). These findings suggest that the D372H variant predominantly reduces current amplitude rather than altering channel gating. To test whether the reduction in current density in the WT+D372H (1:1) group exceeded that expected from simple haploinsufficiency, we compared the observed current density with the theoretical 50% of WT current. The predicted haploinsufficiency level was −236.55 ± 41.13 pA/pF. The observed current density in WT+D372H cells (−158.54 ± 28.37 pA/pF) was significantly lower than the predicted 50% WT level (one-sample *t*-test vs. predicted value, *p* < 0.01), supporting a dominant-negative effect of the D372H variant (Figure 3E).

### 3.2. Reduced Nav1.5 Expression in Cells Expressing the SCN5A-D372H Variant

**Figure 4:**
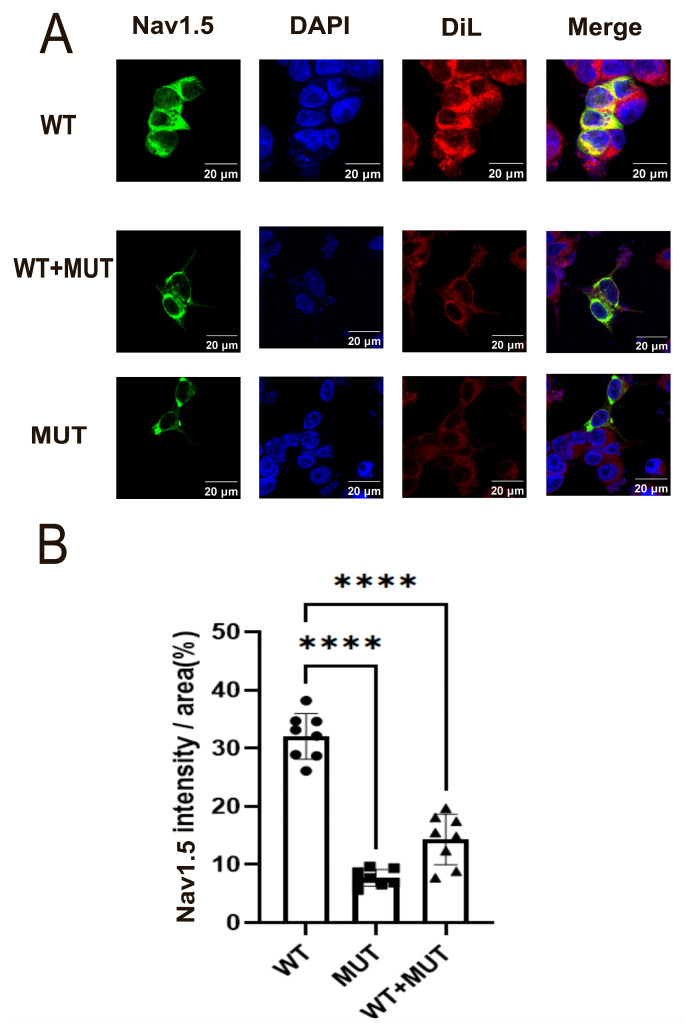
Reduced Nav1.5 expression induced by the *SCN5A* p.D372H variant. (**A**) Representative immunofluorescence images showing Nav1.5 expression in cells transfected with WT, D372H, or WT+D372H (1:1). Nav1.5 is shown in green. DiI (red) was used as a membrane marker, and nuclei were counterstained with DAPI (blue). The D372H mutant and WT+D372H groups exhibited reduced Nav1.5 fluorescence intensity compared with the WT group. (**B**) Quantitative analysis of Nav1.5 fluorescence intensity. Expression levels were significantly decreased in the WT+D372H co-transfection group compared with WT and were lowest in the D372H group. Data are presented as mean ± SD (n = 8 per group). **** *p* < 0.0001.

To further investigate the effect of the *SCN5A*-D372H variant on the functional expression of the NaV1.5 sodium channel protein, confocal microscopy was performed in transfected HEK293 cells. As shown in Figure 4A, Nav1.5-WT displayed strong and concentrated green immunofluorescence, whereas the Nav1.5-D372H mutant and WT+MUT groups exhibited markedly reduced fluorescence intensity. Quantitative immunofluorescence analysis revealed that Nav1.5 fluorescence intensity was highest in the WT group, significantly reduced in the WT+MUT co-transfection group compared with the WT group (*p* < 0.05), and lowest in the MUT group (*p* < 0.05). Notably, the fluorescence intensity in the WT+MUT group was markedly decreased and closer to that observed in the MUT group than in the WT group (Figure 4B).

### 3.3. D372H Reduces SCN5A mRNA Abundance and Stability Independent of Transfection Efficiency

**Figure 5:**
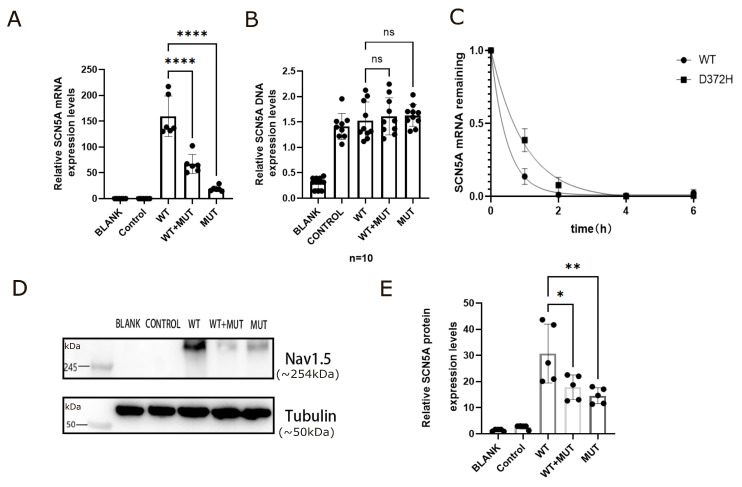
The *SCN5A* p.D372H variant reduces Nav1.5 expression through decreased mRNA abundance and stability, independent of plasmid DNA levels. (**A**) Relative *SCN5A* mRNA expression measured by RT-qPCR. Transcript levels were significantly reduced in the D372H group compared with WT (**** *p* < 0.0001). Co-expression of WT and D372H (WT+MUT) also resulted in a significant decrease in *SCN5A* mRNA expression compared with WT (**** *p* < 0.0001). Data were normalized to WT. (**B**) Quantification of intracellular *SCN5A* plasmid DNA levels. No significant differences were observed among WT, WT+MUT, and MUT groups (ns), indicating comparable transfection efficiency. (**C**) *SCN5A* mRNA decay following transcriptional inhibition with actinomycin D. Relative mRNA remaining was normalized to the 0-h time point. The D372H group exhibited accelerated mRNA decay compared with WT, indicating reduced transcript stability. (**D**) Representative Western blot images showing total Nav1.5 protein expression in BLANK, CONTROL, WT, WT+MUT, and MUT groups. Molecular weight markers are indicated in kilodaltons (kDa). The predicted molecular weight of native Nav1.5 is approximately 227 kDa; however, in this study *SCN5A* was expressed as a GFP-tagged fusion protein, resulting in an apparent band migrating above 245 kDa. Tubulin (50 kDa) was used as a loading control. (**E**) Quantitative analysis of Nav1.5 protein expression normalized to Tubulin. Protein levels were significantly reduced in the D372H group compared with WT (* *p* < 0.05). The WT+MUT group also showed decreased expression compared with WT (** *p* < 0.01). Data are presented as mean ± SD from at least three independent experiments.

The impact of the *SCN5A*-D372H variant on Nav1.5 expression was first evaluated at the transcript level. RT-qPCR analysis demonstrated that *SCN5A* mRNA expression was highest in the WT group and significantly reduced in cells expressing D372H (**** *p* < 0.0001, Figure 5A). Importantly, the WT+MUT co-transfection group also exhibited a marked decrease in transcript levels compared with WT (**** *p* < 0.0001 Figure 5A), with expression levels substantially lower than the theoretical 50% of WT, suggesting that the mutant allele affects overall transcript abundance rather than acting through simple dilution. To exclude unequal transfection efficiency as a potential confounding factor, intracellular plasmid DNA levels were first quantified across experimental groups. No significant differences were detected among WT, WT+MUT, and MUT conditions (ns, Figure 5B), demonstrating comparable plasmid uptake. These findings indicate that the observed reduction in *SCN5A* mRNA levels is not attributable to differences in plasmid abundance. Having excluded unequal transfection efficiency, we next investigated whether reduced transcript levels were due to impaired mRNA stability. Transcription was inhibited using actinomycin D, and time-course analysis revealed a more rapid decay of *SCN5A* mRNA in D372H-expressing cells compared with WT (Figure 5C). Nonlinear regression fitting confirmed accelerated transcript degradation in the mutant group, indicating that the D372H variant compromises mRNA stability through a post-transcriptional mechanism.

Consistent with the mRNA findings, Western blot analysis demonstrated that total cellular Nav1.5 protein expression was highest in the WT group and markedly reduced in cells expressing D372H (Figure 5D,E). The uncropped full-length Western blot images with molecular weight markers are presented in Supplementary Figures S2–S5. Molecular weight markers are indicated in kilodaltons (kDa). The predicted molecular weight of native Nav1.5 is approximately 227 kDa (Figure 5D). In the present study, *SCN5A* constructs were expressed as GFP-tagged fusion proteins, which increased the apparent molecular weight and contributed to band migration above the 245 kDa marker. Quantitative analysis further showed that protein levels in the WT+MUT group were significantly lower than those in WT (** *p* < 0.01, Figure 5E) and were more comparable to the MUT group than to the expected 50% WT level.

## 4. Discussion

A key strength of this study lies in the comprehensive evaluation of the *SCN5A* p.D372H variant across molecular and electrophysiological levels, providing mechanistic insight beyond simple current reduction. Importantly, *SCN5A* plasmid DNA quantification demonstrated comparable plasmid abundance among WT, WT+MUT, and MUT groups, excluding unequal transfection efficiency as a potential confounder. Despite equivalent plasmid levels, *SCN5A* mRNA expression was significantly decreased in cells expressing D372H, and actinomycin D chase experiments revealed accelerated transcript decay, indicating that the variant impairs mRNA stability and reduces overall transcript abundance. This reduction at the RNA level was paralleled by markedly decreased Nav1.5 protein expression, as confirmed by Western blot and immunofluorescence analyses. Notably, in the WT+MUT co-expression condition, both mRNA and protein levels were substantially lower than the theoretical 50% WT level, suggesting that the mutant allele affects overall channel expression beyond simple haploinsufficiency. Functionally, whole-cell patch-clamp recordings showed near-complete abolition of sodium current in D372H-expressing cells, and co-expression with WT resulted in a reduction in peak current density that fell significantly below the predicted 50% WT value. This observation supports a dominant-negative-like effect under the present experimental conditions. Importantly, voltage-dependent activation, inactivation, and recovery kinetics were not significantly altered, indicating that the predominant defect arises from reduced channel abundance rather than altered gating behavior. Collectively, these findings demonstrate that the p.D372H variant produces a severe loss-of-function phenotype characterized by impaired transcript stability, decreased Nav1.5 expression, and markedly diminished sodium current in vitro. However, it should be noted that these functional and expression defects were characterized in a heterologous HEK293 expression system, which lacks cardiac-specific sodium channel auxiliary subunits and regulatory proteins. Consequently, the trafficking, gating, and regulatory properties of NaV1.5 observed in this model may not fully recapitulate those in native cardiomyocytes in vivo, and further validation in cardiomyocyte-based systems will be necessary to confirm the physiological relevance of these findings

Missense variants in *SCN5A* reduce sodium current through diverse mechanisms, including altered gating kinetics, defective trafficking, protein misfolding, and reduced transcript abundance [18]. Some variants disrupt activation or inactivation kinetics [19], whereas others impair channel maturation and surface delivery, as exemplified by ER-retained variants such as R1432G [20] and D1690N [21]. The pore-forming S5–S6 linker (pore loop) is a structurally sensitive region essential for channel folding and selectivity filter integrity, and variants within this microdomain frequently produce severe loss-of-function phenotypes, supporting its designation as a functional hotspot. The p.D372H variant identified in this study resides within the Domain I pore loop. Comparison with neighboring variants further emphasizes the pathogenic relevance of this region. For instance, the adjacent p.W374G variant causes markedly reduced INa, depolarizing shifts in activation, and impaired trafficking with partial rescue by mexiletine, while truncating variants such as p.E375X (Table 2) similarly result in profound loss-of-function phenotypes due to structural disruption. Despite close spatial proximity, p.D372H exhibits a distinct mechanistic profile. It did not significantly alter voltage-dependent gating but instead caused a marked reduction in Nav1.5 expression driven by decreased mRNA stability and reduced protein abundance. Co-expression experiments demonstrated sodium current reduction exceeding that predicted by simple haploinsufficiency, consistent with a dominant-negative-like effect under experimental conditions. Although impaired transcript stability appears to represent the primary mechanism, whether D372H additionally affects protein folding or intracellular trafficking remains to be determined. Given the structural sensitivity of the pore-loop region, subtle conformational perturbations impacting channel maturation cannot be excluded. Collectively, these findings indicate that the DI pore loop constitutes both a structural vulnerability site and a zone of mechanistic heterogeneity, where closely positioned substitutions may produce loss-of-function phenotypes through distinct molecular pathways.

The functional data presented here also provide evidence relevant to variant interpretation under the ACMG/AMP framework. The marked reduction in sodium current and decreased protein expression are consistent with the PS3 functional criterion. Its location within a functionally important pore-loop region supports PM1, and its rarity in population databases is consistent with PM2. In silico tools further predict a deleterious effect (PP3). Together, these criteria support classification of D372H as likely pathogenic; however, such classification should be interpreted in conjunction with clinical and genetic evidence. Importantly, the present findings should be viewed as mechanistic functional evidence that may assist variant interpretation rather than as confirmatory clinical proof.

From a clinical perspective, severe *SCN5A* loss-of-function variants have been associated with arrhythmic risk in Brugada syndrome and have informed risk stratification strategies. Variants that markedly reduce inward sodium current in vitro, such as D372H, may help inform future studies exploring how severe *SCN5A* loss-of-function variants relate to arrhythmic risk in Brugada syndrome, although this relationship requires confirmation in clinical and in vivo studies. As such, functional characterization of variants like D372H may ultimately help refine genotype–phenotype correlations and guide future studies of substrate-directed therapies.

## 5. Limitations

The results of this study indicate that the *SCN5A*-D372H variant, located in a critical domain of the NaV1.5 sodium channel, leads to functional impairment characterized by reduced sodium current density and decreased channel expression levels, potentially contributing to the pathogenesis of Brugada syndrome. However, several limitations should be noted. Firstly, we utilized HEK293 cells for our experiments, which, although useful for studying ion channel properties through plasmid-mediated overexpression, do not fully replicate the physiological environment of cardiac myocytes. As a result, factors such as polygenic modifiers relevant to Brugada syndrome may not be adequately represented, limiting the applicability of our findings to native cardiac tissues [22]. Secondly, earlier research has demonstrated that the W374G variant results in a decrease in sodium current density, which can be partially recovered with mexiletine (MEX) [18]. This suggests that trafficking defects associated with *SCN5A* variants in the pore-S6 regions of Domains I, III, and IV can be rescued by MEX, whereas variants in Domain II do not exhibit this property. Nevertheless, further investigation is necessary to clarify the potential therapeutic role of MEX in rescuing the sodium current loss associated with the D372H variant in Brugada syndrome.

## 6. Conclusions

In conclusion, our study demonstrates that the *SCN5A* p.D372H variant markedly impairs Nav1.5 channel function and expression in vitro. These findings provide mechanistic insight and support a potential role of this variant in altering sodium channel function, which may be relevant to Brugada syndrome, though further clinical and in vivo studies are required to confirm its pathogenic role. These findings underscore the importance of further research to explore therapeutic strategies that could mitigate the functional deficits associated with this and similar variants.

## Figures and Tables

**Table 1:** Electrophysiological properties of Nav1.5 channels in HEK293 cells.

Group (n = 8)	Peak INa Density at −20 mV (pA/pF)	Activation *V*_1/2_ (mV)	Activation *κ* (mV)	Inactivation *V*_1/2_ (mV)	Inactivation *κ* (mV)	Recovery τ (ms)
WT	−473.10 ± 82.27	−50.69 ± 5.21	4.37 ± 1.23	−79.32 ± 2.04	−8.37 ± 0.33	0.91 ± 0.11
WT+MUT (1:1)	−158.54 ± 28.37 *	−47.39 ± 8.61	5.06 ± 1.99	−77.27 ± 1.13	−8.78 ± 0.28	0.85 ± 0.10

* *p* < 0.05 vs. *SCN5A*-WT. Abbreviations: *V*_1/2_, half-activation or inactivation voltage; *κ*, slope factor of the Boltzmann fit; *τ*, time constant of recovery from inactivation.

**Table 2:** Functional and Clinical Characteristics of *SCN5A* Variants Located in the Domain I Pore-Loop Region.

Variant	Location (Domain I)	Functional Phenotype (INa)	Trafficking Defect	Pharmacological Rescue	Clinical Phenotype	Reference
p.D372H	DI pore loop (S5–S6)	Near-complete loss of INa	Yes (reduced membrane expression)	Not tested (proposed)	Type 1 Brugada ECG, syncope, AV block	This study
p.W374G	DI pore loop (S5–S6)	Reduced INa + depolarized activation	Yes	Partial rescue by mexiletine	Severe Brugada phenotype	[18]
p.N406S	DI S6 segment	Reduced INa + depolar-ized activation	Not primarily trafficking	Paradoxical response to lidocaine	Brugada syndrome	[19]

## Data Availability

The data presented in this study are available on request from the corresponding author.

## Supplementary Materials

The following supporting information can be downloaded at: https://www.mdpi.com/article/10.3390/biomedicines14030582/s1, Figure S1: Representative schematic traces and voltage-clamp protocols illustrating sodium channel activation, steady-state inactivation, and recovery from inactivation under normal conditions. Figure S2–S5: Supplementary Western blot analyses with molecular weight markers indicated in kilodaltons (kDa). Full-length blots are provided.
