# Supplementary material for: Functional Characterization of the SCN5A p.D372H Variant Associated with Brugada Syndrome
Source: Biomedicines. 2026 Mar 5;14(3):582. doi: 10.3390/biomedicines14030582 (PMC13024415; doi:10.3390/biomedicines14030582)
Supplement: Supplementary file 1 [file biomedicines-14-00582-s001.zip › biomedicines-4146578-supplementary.pdf]

1. Representative voltage-clamp protocols for assessing sodium channel gating properties.

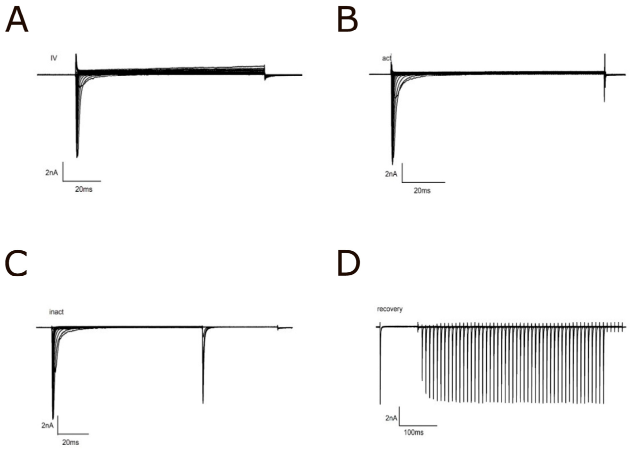

Figure S1. Representative current traces illustrating the voltage-clamp protocols used to assess sodium channel activation (A), steady-state inactivation (B–C), and recovery from inactivation (D).

2 . Western Blot

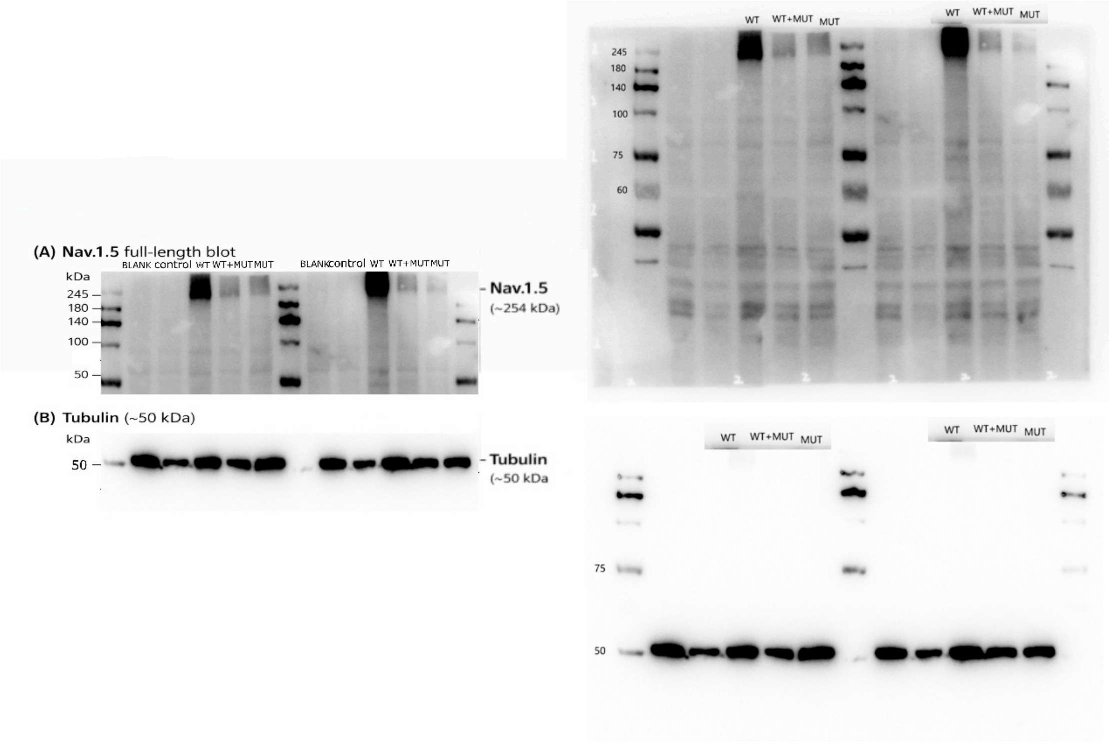

Supplementary Figure S2. Western blot analysis of Nav1.5 expression in HEK293 cells. HEK293 cells were transfected with SCN5A-WT, SCN5A-D372H (MUT), or co-transfected with WT+MUT constructs. Total protein extracts were subjected to SDS–PAGE and immunoblotting using anti-Nav1.5 antibody. The expected molecular weight of the Nav1.5-GFP fusion protein is approximately 254 kDa. Tubulin (~50 kDa) was used as a loading control. Molecular weight markers are indicated in kDa.

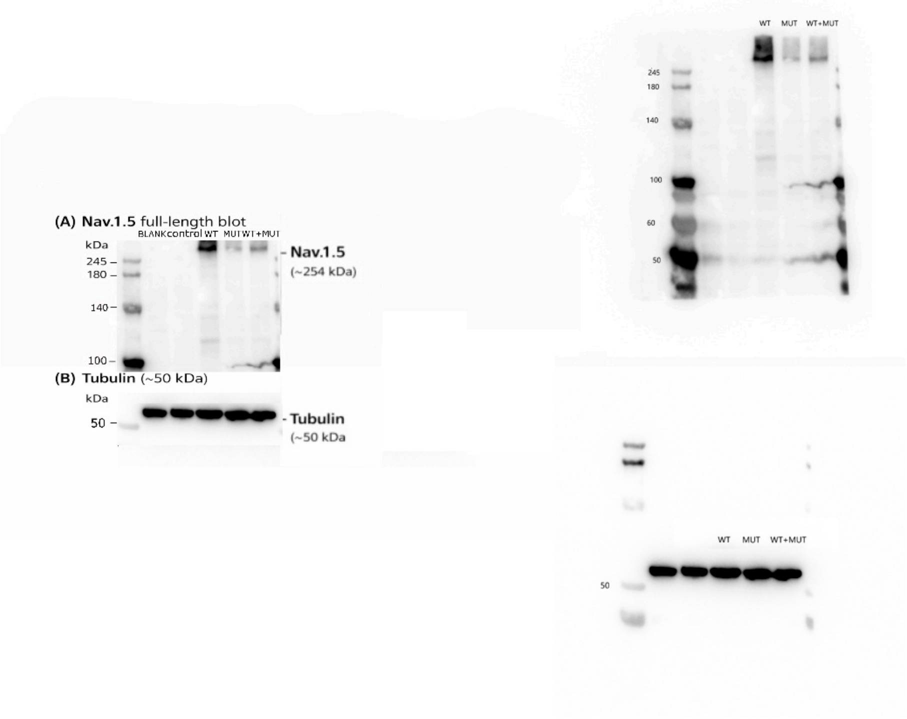

Supplementary Figure S3. Western blot analysis of Nav1.5 expression in HEK293 cells. HEK293 cells were transfected with SCN5A-WT, SCN5A-D372H (MUT), or co-transfected with WT+MUT constructs. Total protein extracts were subjected to SDS–PAGE and immunoblotting using anti-Nav1.5 antibody. The expected molecular weight of the Nav1.5-GFP fusion protein is approximately 254 kDa. Tubulin (~50 kDa) was used as a loading control. Molecular weight markers are indicated in kDa.

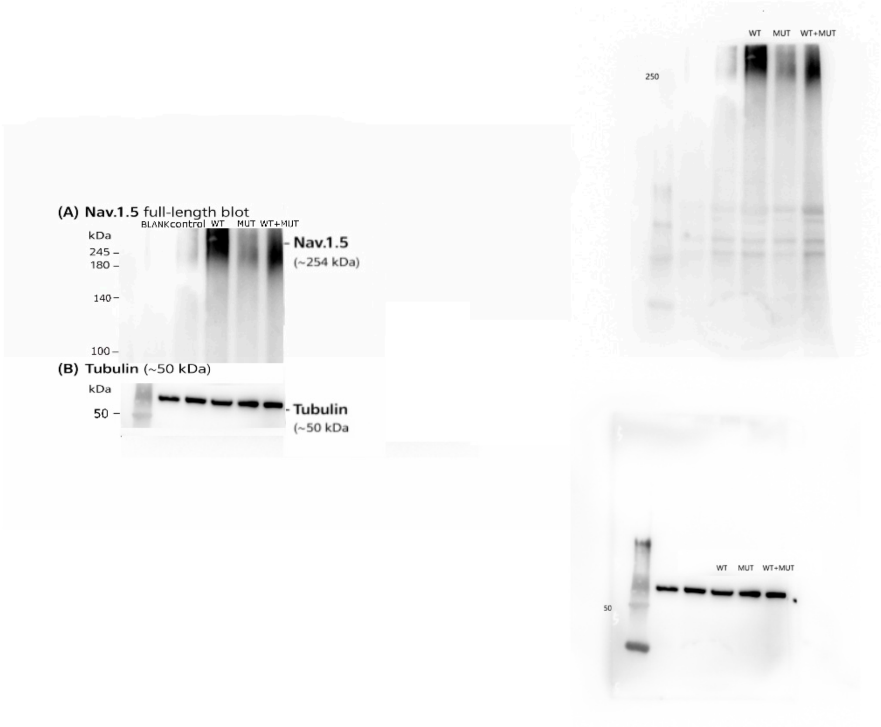

Supplementary Figure S4. Western blot analysis of Nav1.5 expression in HEK293 cells. HEK293 cells were transfected with SCN5A-WT, SCN5A-D372H (MUT), or co-transfected with WT+MUT constructs. Total protein extracts were subjected to SDS–PAGE and immunoblotting using anti-Nav1.5 antibody. The expected molecular weight of the Nav1.5-GFP fusion protein is approximately 254 kDa. Tubulin (~50 kDa) was used as a loading control. Molecular weight markers are indicated in kDa.

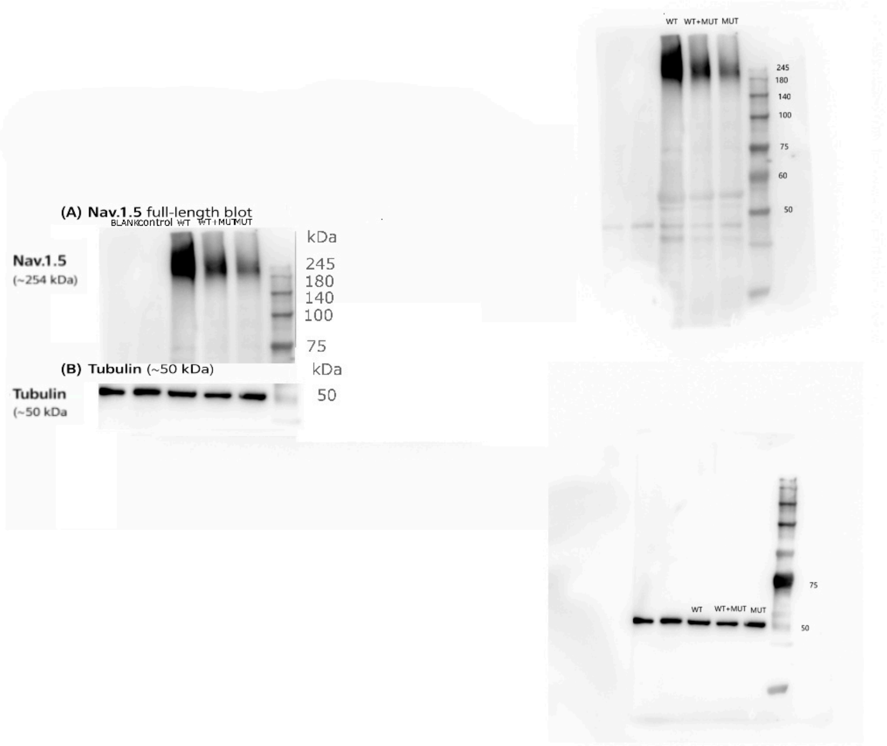

Supplementary Figure S5. Western blot analysis of Nav1.5 expression in HEK293 cells. HEK293 cells were transfected with SCN5A-WT, SCN5A-D372H (MUT), or co-transfected with WT+MUT constructs. Total protein extracts were subjected to SDS–PAGE and immunoblotting using anti-Nav1.5 antibody. The expected molecular weight of the Nav1.5-GFP fusion protein is approximately 254 kDa. Tubulin (~50 kDa) was used as a loading control. Molecular weight markers are indicated in kDa.
